# Enhanced Dopamine in Prodromal Schizophrenia (EDiPS): a new animal model of relevance to schizophrenia

**DOI:** 10.1038/s41537-019-0074-z

**Published:** 2019-03-29

**Authors:** Alice Petty, Xiaoying Cui, Yasvir Tesiram, Deniz Kirik, Oliver Howes, Darryl Eyles

**Affiliations:** 1 Queensland Brain Institute, University of Queensland, Brisbane, QLD 4072 Australia; 2 Centre for Advanced Imaging, University of Queensland, QLD Brisbane, 4072 Australia; 3 BRAINS Unit, Department of Experimental Medical Science, Lund University, 22184 Lund, Sweden; 4 Department of Psychosis Studies, Institute of Psychiatry, Psychology & Neuroscience, King’s College London, London, UK; 5 MRS London Institute of Medical Sciences, Hammersmith Hospital, London, UK; 6 Institute of Clinical Sciences, Faculty of Medicine, Imperial College London, London, UK; 7 Queensland Centre for Mental Health Research, Wacol, QLD 4076 Australia

## Abstract

One of the most robust neurochemical abnormalities reported in patients living with schizophrenia is an increase in dopamine (DA) synthesis and release in the dorsal striatum (DS). Importantly, it appears that this increase progresses as a patient transitions from a prodromal stage to the clinical diagnosis of schizophrenia. Here we have recreated this pathophysiology in an animal model by increasing the capacity for DA synthesis preferentially within the DS. To achieve this we administer a genetic construct containing the rate-limiting enzymes in DA synthesis—tyrosine hydroxylase (TH), and GTP cyclohydrolase 1 (GCH1) (packaged within an adeno-associated virus)—into the substantia nigra pars compacta (SNpc) of adolescent animals. We refer to this model as “Enhanced Dopamine in Prodromal Schizophrenia” (EDiPS). We first confirmed that the TH enzyme is preferentially increased in the DS. As adults, EDiPS animals release significantly more DA in the DS following a low dose of amphetamine (AMPH), have increased AMPH-induced hyperlocomotion and show deficits in pre-pulse inhibition (PPI). The glutamatergic response to AMPH is also altered, again in the DS. EDiPS represents an ideal experimental platform to (a) understand how a preferential increase in DA synthesis capacity in the DS relates to “positive” symptoms in schizophrenia; (b) understand how manipulation of DS DA may influence other neurotransmitter systems shown to be altered in patients with schizophrenia; (c) allow researchers to follow an “at risk”-like disease course from adolescence to adulthood; and (d) ultimately allow trials of putative prophylactic agents to prevent disease onset in vulnerable populations.

## Introduction

An abnormality in dopamine (DA) signalling has endured as one of the most robust hypotheses for the neurobiology of schizophrenia. The latest version of this hypothesis highlights the importance of pre-synaptic DA abnormalities. Early support for this hypothesis came from positron emission tomography (PET) studies using low dose amphetamine (AMPH), showing that patients had increased DA release by assessing the displacement of ligands specific for striatal D2 receptors.^1–3^ Further confirmation came from PET studies in patients using radiolabelled *l-*dihydroxyphenylalanine (*l-*DOPA, the precursor to DA). Two different meta-analyses confirmed that patients have increased uptake of radiolabelled *l*-DOPA and increased DA release in the striatum.^4,5^ Increased uptake of *l*-DOPA is believed to reflect increased DA synthesis, since *l*-DOPA conversion to DA via amino acid decarboxylase (AADC) is not rate limited.^6^ Of those PET studies (seven in total) that examined striatal subdivisions, this finding was restricted to the associative or dorsal striatum (DS).^7^ It is also emerging that increased *l*-DOPA uptake in the associative striatum is associated with treatment responsiveness in patients.^8^ Although a number of PET studies have also examined DA transporter availability and the binding potential of radiotracers to D2/3 receptors, no consistent abnormalities have been found in patients with schizophrenia.^5,9,10^

The prodrome of schizophrenia is a period prior to the full disease, in which patients show attenuated symptoms. In order to clinically assess the prodrome, it has been necessary to identify people with attenuated symptoms through such tools as the Comprehensive Assessment of At-Risk Mental States (CAARMS), which identifies those at high risk of developing schizophrenia in the following year or so.^11^ These attenuated symptoms include infrequent and/or mild hallucinations and delusions, but also muted negative symptoms and cognitive deficits.^12,13^ For ~20% of these high risk individuals, symptoms will become progressively worse until transition to the clinical diagnosis of schizophrenia.^14^ Interestingly, the increased uptake of radiolabelled *l*-DOPA in the DS seen in clinical schizophrenia is also present in people meeting CAARMS and similar criteria for being at high risk of developing schizophrenia.^15–17^ Moreover, longitudinal studies indicate that the elevation in *l*-DOPA uptake is most marked in people who go on to schizophrenia (i.e. those in the prodromal phase as opposed to those who meet high risk criteria but don’t go on to develop schizophrenia in the next year),^18^ and there is a further increase in *l*-DOPA uptake with disease progression.^19^ Importantly, these findings suggest that intervention in this process may represent a prophylactic target. Understanding the neurochemical alterations that are responsible for the progression from prodrome to clinical schizophrenia is crucial in this endeavour. It is important to note that, since in EDiPS animals we seek to replicate the neurobiology seen in those patients who do progress to schizophrenia, this is a model of the prodrome, rather than of the entire “at-risk” population.

There are indications that the glutamatergic system is also altered in schizophrenia. A meta-analysis of ^1^H-MRS studies in schizophrenia patients demonstrated increased glutamate (Glu) and glutamate + glutamine (Glx) in the basal ganglia of patients.^20^ Additional studies have shown that this increase is prominent in the DS,^21,22^ however there are indications that medication status and disease chronicity can dramatically alter these glutamatergic outcomes.^23^ While there is variability within these results, understanding the connection between the dopaminergic and glutamatergic systems in the DS in patients with schizophrenia could be pivotal in understanding the prominent negative symptoms and cognitive deficits seen in patients.

**Fig. 1:**
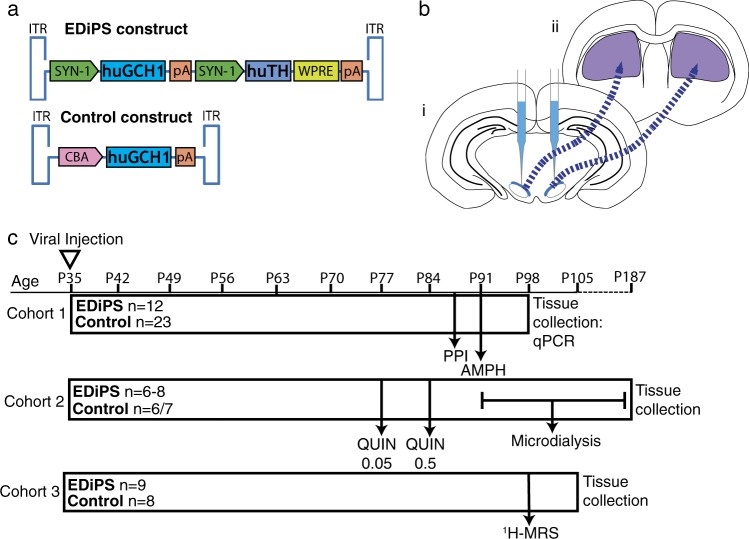
Outline of the model. **a** EDiPS and Control genetic constructs. **b** Schematic illustration of injection target and projections. The packaged construct is injected bilaterally into the substantia nigra pars compact (SNpc) (i)—blue. Cell bodies from this region project preferentially to the DS (ii)—purple. **c** Timeline of injections and tests in the three different cohorts. All injections were performed at post-natal day (P) 35. For cohort 1, PPI was performed ~7.5 weeks following injection of the construct, and AMPH-induced locomotion was tested the following day. Tissue was collected one week later. For cohort 2, animals were given 0.05 mg/kg quinpirole, then the 0.5 mg/kg dose 1 week later. One week following the final quinpirole test, microdialysis was performed, and tissue was collected immediately. For cohort 3, ^1^H-MRS was performed approximately 8 weeks following construct injection, and tissue was collected 1 week later. ITR: inverted terminal repeat, SYN-1: Synapsin 1, huGCH1: human GTP cyclohydrolase 1, pA: polyadenylation tail, huTH: human tyrosine hydroxylase, WPRE: Woodchuck Hepatitis Virus Post-transcriptional Response Element, CBA: Chicken Beta-Actin promoter, PPI: pre-pulse inhibition, AMPH: amphetamine, QUIN: quinpirole, ^1^H-MRS: proton magnetic resonance spectroscopy

Many animal models of schizophrenia exist, however none so far have replicated the widely reported increase in DA synthesis and release in the DS seen in patients. In order to establish such a model, we have employed a genetic construct coding for tyrosine hydroxylase (TH) and GTP cyclohydrolase 1 (GCH1), which are rate-limiting enzymes in the synthesis of DA (Fig. 1a).^24^ Prior work has established that the use of these enzymes in combination results in higher levels of DA synthesis compared to TH alone.^25^ This work also established that GCH1 alone has no effect on DA synthesis, since even in the precense of excess tetrahydrobiopterin (as a result of increased GCH1), TH remains the limiting factor in the conversion of tyrosine to DOPA. We packaged this construct into an adeno-associated viral (AAV) vector. By delivering this construct stereotaxically to the substantia nigra pars compacta (SNpc) we achieve spatial selectivity, since the primary dopaminergic inputs to the DS in humans and rodents come from the SNpc^26,27^ (Fig. 1b). The construct is injected bilaterally at a period equivalent to early adolescence in a rodent (post-natal day 35) to potentially mimic the schizophrenia prodrome. We refer to this model as “Enhanced Dopamine in the Prodrome of Schizophrenia” (EDiPS).

The primary aim of this project was to produce an animal model which shows enhanced DA transmission preferentially in the DS. Our second aim was to assess the expression of well-described behavioural phenotypes in EDiPS that have relevance to the positive symptoms of schizophrenia and other psychiatric conditions, such as psychosis in bipolar disorder, where elevated *l*-DOPA uptake has also been reported.^28^ The final aim was to explore whether inducing such an alteration led to changes in other neurotransmitters, both at baseline and following AMPH. Our findings suggest that this new pathophysiologically-based model will be useful in studying how increased pre-synaptic DA synthesis and release in the DS might result in the progression towards schizophrenia.

## Results

### Unilateral proof-of-concept experiment

**Fig. 2:**
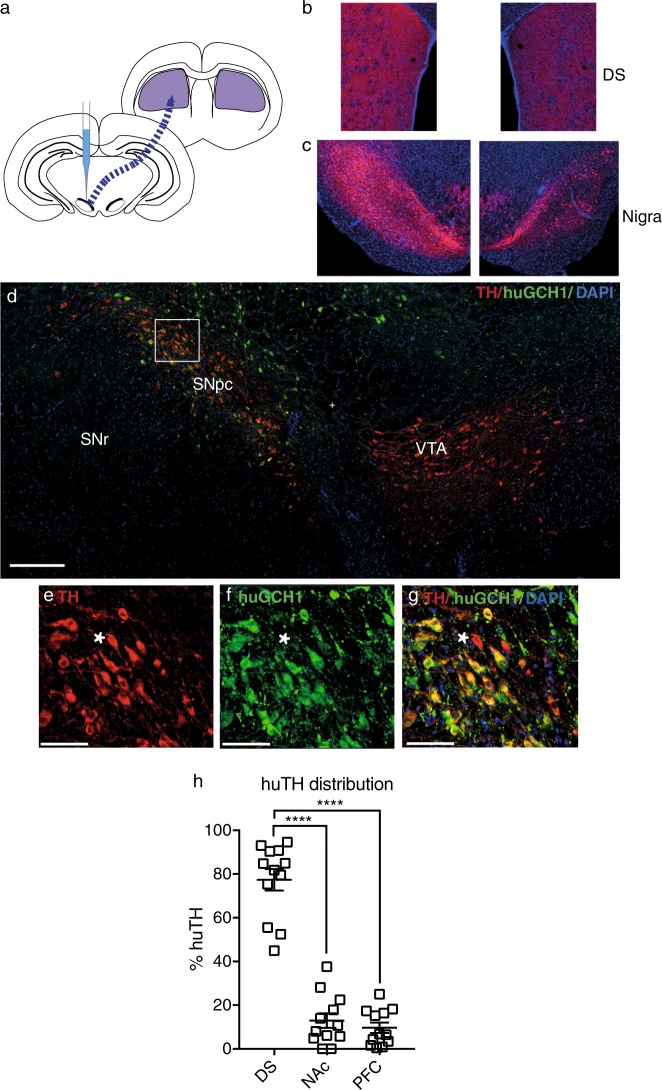
Distribution of the EDiPS construct. As a proof-of-concept, a small group of animals were injected unilaterally with the EDiPS construct (**a**). The dorsal striatum (DS) and nigral regions were immunostained for tyrosine hydroxylase (TH). The midbrain showed dramatically increased TH staining in the injected hemisphere (**c**, left) compared to the non-injected hemisphere (**c**, right). TH staining was also more pronounced in the DS of the injected hemisphere (**b**, left) compared to the non-injected hemisphere (**b**, right). **d** Shows a representative EDiPS animal midbrain stained for huGCH1 and TH. HuGCH1 (reflecting construct expression) was prominant in the substantia nigra pars compacta (SNpc) and in adjacent cells but largely absent from the ventral tegmental area (VTA). The inset within the pars compacta (**e**–**g**) shows a number of TH+/huGCH1+ co-labelled cells indicating infected dopaminergic cells, and an example of a TH+/huGCH1- (non-infected) cell (star). SNr: substantia nigra pars reticulata. **h** Shows the proportion of huTH mRNA in each projection target region for the 12 EDiPS animals from cohort 1. Scale bar: Fig. 2d = 300μm, 2e–g = 80μm. For statistical analysis, see text. ****p < 0.0001 (ANOVA). ±SEM

In the unilaterally injected animals (Fig. 2a), there was a clear increase in TH staining in the injected (left) hemisphere both at the midbrain injection site (Fig. 2c), and in the DS (Fig. 2b). The antibody used to visualize huGCH1 does not cross-react with endogenous ratGCH1,^29^ and therefore indicates the spread of the construct in the midbrain (Fig. 2d–g). HuGCH1 staining was assessed in cohort 1 and was visible in every EDiPS animal in at least 1 hemisphere (Table S2). HuGCH1 was also present in animals that received the control construct, but was absent in the vehicle treated animals.

### Distribution of huTH mRNA in the dorsal striatum

The localization of human (construct-generated) TH mRNA was assessed in the projection targets of midbrain DA neurons; the DS, nucleus accumbens (NAc) and pre-frontal cortex (PFC). An ANOVA revealed that there was a significant difference between levels of huTH mRNA in these regions (*F*_(2,33)_ = 70.8, *p* = < 0.0001) (Fig. 2h). Post hoc analyses revealed significantly higher levels of huTH mRNA in the DS (78%) compared to the NAc (12%; *p* < 0.0001; *t*-test) and PFC (10%; *p* < 0.0001; *t*-test). No huTH mRNA was detected in either the control or vehicle animals.

### Effect of EDiPS construct on expression of DA-related genes

There were no significant differences between EDiPS and control animals for any gene measured in any region (Table S2; MANOVA). There was also no effect of huTH on endogenous ratTH expression in EDiPS animals (Fig. S1).

### Microdialysis neurochemical assessments

**Fig. 3:**
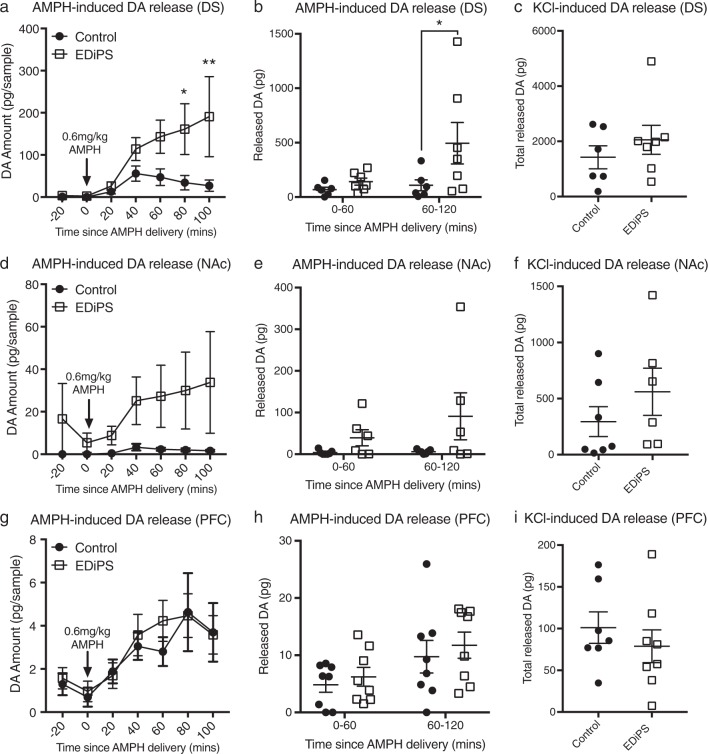
AMPH- and KCl-induced dopamine (DA) release. Following baseline, amphetamine (AMPH) is delivered i.p. at the start of the first AMPH timepoint (T0), and 6 AMPH timepoints are collected. Each timepoint represents a 20-min bin. The graphs in (**a**, **d**, **g**) show data separated into 20-min bins, to represent the timecourse of DA release following AMPH, while (**b**, **e**, **h**) represent the bins pooled for the first hour and second hour following AMPH administration. AMPH-induced DA release was significantly increased during the second hour following AMPH in the dorsal striatum (DS) (**b**), but not in the nucleus accumbens (NAc) (**e**), or pre-frontal cortex (PFC) (**h**). The amount of DA released by KCl infusion (over 1 h) was not significantly different between control and EDiPS animals for the DS (**c**), NAc (**f**) or PFC (**i**). For statistical analysis, see text. *p < 0.05, **p < 0.01 (RM ANOVA, two-way ANOVA). DS *n* = 6 (control), *n* = 7 (EDiPS); NAc *n* = 7 (control), *n* = 6 (EDiPS); PFC *n* = 7 (control), *n* = 8 (EDiPS). ±SEM

When DA release in response to AMPH was assessed with a repeat measures (RM) ANOVA, there was a time*EDiPS interaction in the DS (*F*_(6,66)_ = 2.274, *p* = 0.047) (Fig. 3a). Post-hoc analysis revealed this increase in AMPH-induced DA release to be significant at 80 minutes (*p* = 0.02) and 100 minutes post-AMPH (*p* = 0.004). When data was pooled into one-hour bins following AMPH administration, EDiPS animals showed significantly increased DA released in the second hour following AMPH (*t*_(22)_ = 2.53, *p* = 0.037; *t*-test with correction for multiple comparisons) (Fig. 3b). This increase was not significant in the NAc (*F*_(6,66)_ = 1.85, *p* = 0.10, *t*_(22)_ = 2.19, *p* = 0.07) (Fig. 3d, e) or the PFC (*F*_(6,66)_ = 0.19, *p* = 0.97, *t*_(22)_ = 0.67, *p* > 0.99) (Fig. 3g, h). There were no effects of EDiPS on AMPH-induced changes for homovanillic acid (HVA), 3-methoxytyramine (3-MT), or 5-hydroxyindoleacetic acid (5-HIAA) (Figs. S2 and S3). A 2-way ANOVA was conducted on total DA released by KCl over a one-hour period. There was a significant effect of brain region on KCl-released DA (*F*_(2,36)_ = 19.04, *p* < 0.001), but no effect of EDiPS (*F*_(1,36)_ = 1.61, *p* = 0.21) or a region*EDiPS interaction (*F*_(2,36)_ = 0.69, *p* = 0.51) (Fig. 3c, f, i). Semi-quantitative immunohistochemical analysis was performed to determine whether any correlations were present between construct expression in the midbrain, and AMPH- or KCl-induced DA release in either the DS or NAc (Supplementary methods and Fig S4). No significant correlations were evident (Pearson’s *r*). There was also no correlation between AMPH- and KCl-induced DA release in the DS or NAc for either group (Fig. S5).

When assessing baseline levels of each neurotransmitter, the average of the three 20-min baseline samples was used to generate the data shown in Table 1. A MANOVA was used to examine the effect of region and group (EDiPS vs control) for all analytes. The 5-HT metabolite 5-HIAA was decreased in EDiPS animals across all three regions (*F*_(1,35)_ = 5.79, *p* = 0.021), but there were no other differences in baseline neurotransmitters. For additional assessments of DOPAC, HVA, 3-MT, 5-HT and 5-HIAA, see figures S2 and S3.

**Table 1:** Dopamine, serotonin and metabolites (dialysate) pg/sample

Analyte	DS		NAc		PFC	
	Control	EDiPS	Control	EDiPS	Control	EDiPS
DA	0.59 (0.41)	2.13 (0.71)	n.d.	n.d.	1.39 (0.41)	1.53 (0.35)
DOPAC	5445 (1283)	3300 (618)	3034 (738.1)	2855 (596.9)	303.7 (157.2)	108.8 (21)
HVA	4308 (631)	4202 (1444)	2565 (419.3)	2254 (469.1)	574.4 (196.4)	586 (224.7)
3-MT	45.1 (29.9)	109.5 (53.3)	56.3 (30.7)	97.6 (51.5)	79.3 (47.5)	339.7 (140.8)
5-HT	7.6 (3.5)	21.9 (4.9)	5.46 (2.8)	32.8 (30.9)	62.0 (24.1)	161.3 (90.07)
5-HIAA	1186 (173.9)	909.1 (102.4)^a^	1389 (229.6)	1095 (62.5)^a^	1679 (292.2)	1144 (137.3)^a^

Baseline levels of microdialysis analytes. These values represent the average value of the three baseline samples. Mean (SEM).

*DOPAC* 3,4-dihydroxyphenylacetic acid, *HVA* homovanillic acid, *3-MT* 3-methoxytyramine, *5-HT* 5-hydroxytryptophan (serotonin), *5-HIAA* 5-hydroxyindoleacetic acid

^a^Group difference across all regions

### Behaviour

There was no significant difference between the animals receiving the control construct or saline vehicle for any behavioural tests (Fig. S6; ANOVA), therefore these groups were pooled.

### AMPH-induced locomotion

**Fig. 4:**
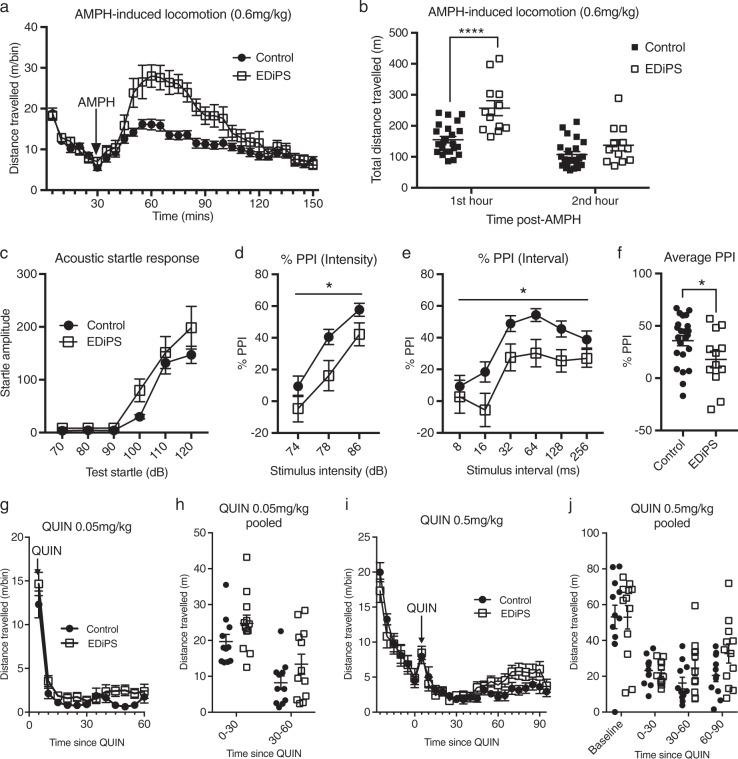
Behavioural assessment of EDiPS animals. **a** All animals habituated normally, and showed increased locomotion following the amphetamine (AMPH) i.p. injection at 30 minutes. **b** EDiPS animals showed significantly increased total locomotion in the first hour following AMPH administration. **c** EDiPS animals showed a normal acoustic startle response (ASR). EDiPS animals showed decreased %PPI across different pre-pulse intensities **d** and pre-pulse intervals **e**. EDiPS animals showed significantly decreased % pre-pulse inhibition (PPI) across all trials **f** compared to control animals. There was no difference between groups for the locomotor response to 0.05 mg/kg quinpirole (QUIN) **g**, **h** or 0.5 mg/kg quinpirole **i**, **j**. For statistical analysis, see text. *p < 0.05, ****p < 0.0001 (RM ANOVA, *t*-test). AMPH-induced locomotion and PPI: *n* = 23 (control), *n* = 12 (EDiPS). Quinpirole locomotion: *n* = 9 (control), *n* = 8 (EDiPS). Coefficient of variation for **b** Control = 35.3%, EDiPS = 33.8%. ±SEM

During the first 30 min of the test, both EDiPS and control animals showed typical habituation to the chamber (Fig. 4a). There was a significant effect of group on total locomotion following AMPH injection (*F*_(1,33)_ = 11.6, *p* = 0.0017; RM ANOVA), with EDiPS animals moving significantly more in the first hour compared to control animals (*p* < 0.0001; *t*-test) (Fig. 4b).

### Pre-pulse inhibition (PPI)

Both EDiPS and control animals showed a normal increase in startle response amplitude with increasing pulse intensity (*F*_(1,33)_ = 2.6, *p* = 0.11; RM ANOVA) (Fig. 4c). However there was a main effect of treatment group for %PPI across different pre-pulse intensities (*F*_(1,33)_ = 4.178, *p* = 0.05; RM ANOVA) (Fig. 4d) and pre-pulse intervals (*F*_(1,33)_ = 4.2, *p* = 0.049; RM ANOVA) (Fig. 4e). When %PPI was pooled for all pre-pulse intensities and intervals, EDiPS animals showed significantly impaired %PPI compared to control animals (*t*_(33)_ = 2.0, *p* = 0.049; *t*-test) (Fig. 4f).

### Quinpirole-induced locomotion

Analysis of the data pooled into 30-min time bins revealed that there were no significant differences between control and EDiPS animals in total locomotor activity following either the 0.05 mg/kg (*F*_(1,21)_ = 3.6, *p* = 0.07; RM ANOVA) (Fig. 4g, h) or 0.5 mg/kg (*F*_(1,21)_ = 2.1, *p* = 0.16; RM ANOVA) (Fig. 4i, j) dose of quinpirole.

### ^1^H-MRS analysis

**Fig. 5:**
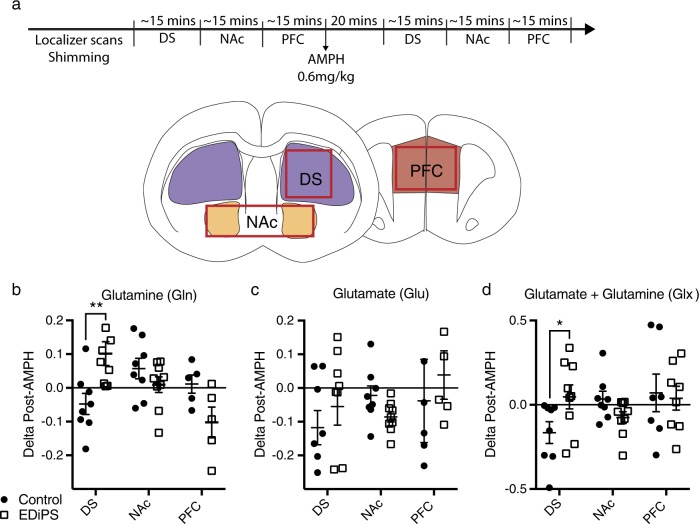
^1^H-MRS analysis of EDiPS animals. **a** The proton magnetic resonance spectroscopy (^1^H-MRS) scanning protocol begins with localizer scans and shimming processes. Then each region is scanned in sequence, which takes approximately 15 min for each region. AMPH is then delivered i.p., and 20 min later, each region is scanned again in the same sequence. The dorsal striatum (DS) voxel was unilateral, whereas the nucleus accumbens (NAc) and pre-frontal cortex (PFC) voxels were bilateral. The delta scores for post-pre-AMPH values were examined for each analyte across regions. There was a significant difference in delta values for Glutamine (Gln) **b** and Glutamate + Glutamine (Glx) **d** but not Glutamate (Glu) **c**. Posthoc analyses revealed that the statistical differences were confined to the DS for both Gln and Glx. For full statistical analysis, see text. *p < 0.05, **p < 0.01. (two-way ANOVA). *n* = 8 (Control), *n* = 9 (EDiPS). ±SEM

There was no effect of EDiPS on the baseline levels of any analyte (Table S4; MANOVA). There was a significant region*EDiPS interaction for the post-AMPH change in glutamine (Gln) (*F*_(2,38)_ = 8.559, *p* = 0.001; two-way ANOVA) (Fig. 5b), and post-hoc analysis revealed a significant difference only in the DS (*p* = 0.006; *t*-test). There was also a region*EDiPS interaction for the change in Glx post-AMPH (*F*_(2,43)_ = 3.213, *p* *=* 0.049; two-way ANOVA) (Fig. 5d), which again was only significant in the DS (*p* = 0.044; *t*-test). Paired *t*-tests confirmed that Gln was significantly increased post-AMPH in EDiPS animals (*p* = 0.02) but unchanged in controls (*p* = 0.17). Glx was significantly decreased post-AMPH in control animals (*p* = 0.037) but not in EDiPS animals (*p* = 0.52). This finding is likely driven by a decrease in Glu in controls, since there was a trend level decrease of Glu post-AMPH in controls (*p* = 0.053) but not in EDiPS animals (*p* = 0.34) (Fig. 5c). There were no other differences in delta values (Table S4).

## Discussion

EDiPS was developed to replicate arguably the most robust neurochemical finding in patients with schizophrenia, namely increased pre-synaptic DA levels in the DS. EDiPS achieves this by increasing DA synthesis capacity preferentially in the rat DS. The presence of huTH did not induce a baseline increase in DA levels within the DS. However, EDiPS animals show a significant increase in AMPH-induced DA release in the DS. Interestingly, control animals show the typical peak in DA release around 60 minutes post-injection, where DA levels in EDiPS animals continue to rise over the 2 hour collection period. This finding could be the result of a deficit of the DA transporter (DAT) at clearing synaptic DA, however mRNA levels of DAT are normal in EDiPS animals in all regions (Table S2). Whilst this does not discount the possibility of a change in DAT activity or function, it suggests that this is not the primary mechanism explaining this finding. Early studies have shown that AMPH stimulates the synthesis of DA, and selectively releases newly-synthesized DA.^30–32^ EDiPS animals have increased capacity to synthesise DA within the DS. This may be the reason for the prolonged kinetics of DA release in the DS of EDiPS animals. To some extent this conclusion is supported by the finding that KCl-mediated DA release in EDiPS animals is normal, indicating that the stored vesicular pool of DA is unaltered in these animals.

There is evidence that patients with schizophrenia show increased baseline levels of DA^33^ as well as an enhanced response to low doses of AMPH.^1,34,35^ EDiPS animals replicate this with increased DA release in response to a low AMPH dose. This alteration in subcortical DA is primarily evident in the DS. This animal model therefore replicates the core dopaminergic pathophysiology repeatedly observed in patients with schizophrenia. It would be intriguing to examine whether patients with schizophrenia also have altered DA release kinetics in response to AMPH, however PET studies are rarely conducted over the longer time frames required for such a study.

EDiPS animals also clearly recapitulate behavioural phenotypes of relevance to the “positive” symptoms of schizophrenia; increased AMPH-induced hyperlocomotion and deficits in PPI. Increased hyperlocomotion in rodents following AMPH is widely interpreted to indicate increased subcortical DA release. Similarly, deficits in PPI have long been considered an endophenotype of schizophrenia.^36^ Further work is necessary to identify whether EDiPS animals demonstrate any behaviours relevant to the negative symptoms or cognitive impairments seen in patients.

AMPH-mediated locomotion is thought to be under dopaminergic control largely within the NAc.^37,38^ However, there is also some evidence that D1-expressing medium spiny neurons in the DS are crucial for DA-mediated hyperactivity.^39,40^ EDiPS may therefore be useful in understanding the role of DS DA in locomotion. PPI is modulated by a range of brain regions including the NAc, PFC, ventral tegmental area (VTA), and substantia nigra pars reticulata (SNr).^41^ However the DS is also implicated in PPI. For instance, a lesion of the SNpc-DS projection results in impaired PPI.^42^ A very recent study has also demonstrated that infusion of the D1-like antagonist SCH23390 into the DS impairs PPI in rats.^43^ Although we found no difference in mRNA expression for D1 or D2 receptors in the NAc or the DS, these findings do not exclude the possibility that an alteration in D1 affinity, or the balance of D1 and D2 signalling in the DS, might contribute to the behavioural phenotypes seen here.

Although our focus was to replicate the clinical findings in the DS in patients with schizophrenia, DA is also synthesized in the cell bodies found in the SNpc, where the EDiPS construct is injected. DA can be released locally in the midbrain to modulate DA neuron firing properties.^44^ Although unconfirmed, it is possible that we may also have altered somato-dendritic DA signalling in the nigra. Interestingly, a number of in vivo imaging studies suggest that increased DA activity in the midbrain may be a component of schizophrenia pathophysiology.^45–47^ This animal model may also therefore be ideally placed to understand the role of increased nigral dopaminergic function in schizophrenia phenotypes.

The primary 5-HT metabolite, 5-HIAA, was decreased across all brain regions in EDiPS animals. There are also some indications of increased levels of 5-HT across all regons, but most robustly in the DS (Fig. S3). This pattern suggests either decreased 5-HT turnover (although MAO-A mRNA levels were unchanged, see Table S2) or increased 5-HT release across the brain. The dorsal raphe nuclei (DRN) represents the primary source of serotonergic innervation to many regions of the brain. There is evidence that stimulation of the D2 receptors on 5-HT neurons in the DRN by midbrain DA, via either direct projections, or through volume transmission, increases tonic serotonergic activity.^48–51^ As we expect increased DA in the midbrain of EDiPS animals, this may suggest a mechanism by which serotongeric activity could be increased across the EDiPS brain. The relevance of this serotongergic finding in EDiPS animals to patients with schizophrenia remains to be established.

The selective D2/3 receptor agonist quinpirole was used to examine whether the long-term increased capacity to synthesise DA in EDiPS animals had altered either pre-synaptic or post-synaptic DA receptor function. EDiPS animals displayed a normal locomotor response to both the putatively autoreceptor (0.05 mg/kg)^52^ and post-synaptic (0.5 mg/kg)^53^ D2 selective dose. This finding is corroborated by normal mRNA levels for both the D2Rshort and long isoforms in all brain regions (Table S2). This is also consistent with PET studies of D2 receptor binding in schizophrenia patients which indicate no robust changes in D2/3 receptors in schizophrenia patients.^5^

In order to examine the effects of EDiPS on other major neurotransmitters or metabolites in the brain we employed in vivo pharmaco-^1^H-MRS. Baseline analytes were normal. Following AMPH however, EDiPS animals showed an increase in glutamine (Gln) and unchanged Glx, whereas control animals showed normal Gln, and a decrease in Glx (apparently driven by a decrease in Glu). Consistent with most of our other neurochemical studies, these findings were confined to the DS, suggesting a local effect mediated by EDiPS. However, the impact of AMPH on levels of Glu and Gln in the striatum is not immediately clear. To date we are unaware of any studies in which MRS has been employed to study striatal Glu/Gln responses to an AMPH challenge. However a consideration of other studies that have examined Glu and Gln levels in response to varying doses of AMPH or DA either by dialysis or by examining brain tissue at post mortem are informative.

In keeping with the response we observe in control animals, studies indicate that when DA is infused directly into the striatum via reverse microdialysis, levels of Glu and Gln are reduced.^54^ Studies with moderate doses of AMPH are contradictory. One study has shown that a moderate dose of AMPH (2 mg/kg) also led to a reduction in Glu in the striatum.^55^ Another study appears to show the reverse pattern.^56^ One way that AMPH may diminish Glu release is through released DA stimulating inhibitory D2 receptors located on the terminals of glutamatergic afferents.^57,58^

In contrast, the effects of high-dose AMPH on Glu and Gln seem consistent. Brain homogenate data, which incorporates all cellular compartments, and therefore perhaps better reflects our ^1^H-MRS results, shows that Gln levels increase whilst Glu levels remain normal when a high (30 mg/kg) AMPH dose,^59^ or “binge” levels of methamphetamine are used.^60^ It is possible that at high doses AMPH induces endocytosis of the Glu transporter.^61,62^ In such a circumstance diminished Glu uptake may lead to increased levels of Glu in the synaptic cleft.

Therefore EDiPS animals appear to express a glutamatergic response perhaps reflective of a high dose of AMPH (given their increased capacity to synthesise DA) whereas control animals may respond as expected to a lower AMPH dose. Although this mechanism is still far from clear, manipulation of the DA system in the DS of EDiPS animals has clearly had a downstream local effect on Glu/Gln signalling. Understanding the link between DA and Glu in the DS remains a critical question in the field of schizophrenia research.^63^

### Limitations

As this is the first study using this model there are a number of issues still unexplored. First, it is not known whether EDiPS animals display any negative symptom phenotypes, or any cognitive deficits, such as those apparent in clinical schizophrenia and the prodrome. Second, we currently have no evidence of the longitudinal progression of EDiPS phenotypes. Therefore we cannot yet draw a link between this model, and the development and course of schizophrenia. Third, the current genetic construct employs a synapsin promoter, which confers neuronal but not dopaminergic selectivity. The use of a TH promoter would increase DA neuron selectivity in this model. Fourth, DA systems are still remodelling in adolescence with alterations in DA receptors,^64^ the DAT,^65^ and basal levels of DA.^66^ Therefore adolescence can be considered a vulnerable period in DA system maturation. It is unclear whether the same behavioural phenotypes would be evident if this construct was delivered in adulthood. Finally, the temporal limitations of the ^1^H-MRS protocol means that regions had to be scanned in sequence, rather than simultaneously.

We have established a novel animal model, EDiPS, which replicates the selective increase in DA release in the DS of patients with schizophrenia. This model also produces positive symptom phenotypes. The intriguing findings of glutaminergic abnormalities also suggests that EDiPS could be used to examine the complex interaction between DA and Glu in the DS. Patients “at-risk” of developing schizophrenia show preliminary indications of increased pre-synatic DA uptake/release in the DS, as well as the onset of glutamatergic abnormalities,^67^ and deficits in PPI.^68^ Young adult EDiPS animals display all of these abnormalities. We therefore consider EDiPS to be a unique model in schizophrenia research. Firstly, we replicate one of the most robust neurochemical features of schizophrenia, namely increased DA synthesis and release preferentially within the DS; secondly, we induce this DA abnormality in adolescence, such that EDiPS may represent a useful model to examine the course and progression towards schizophrenia; thirdly, EDiPS appears to also produce glutamatergic abnormalities in the DS which is also observed in “at-risk” patients; and finally, EDiPS could be used to trial prophylactic interventions with the hope of arresting disease progression. We consider that EDiPS has immense potential as a novel animal model of schizophrenia.

## Methods

For detailed methods regarding PPI, tissue collection, RNA extraction etc., and microdialysis surgery, please see supplementary material.

### Animals and housing

Male Sprague-Dawley rats were acquired from the Animal Resources Centre (ARC, South Australia), and pair-housed with ad libitum food and water. Three different cohorts of animals were used (Fig. 1c). All animal procedures were approved by The University of Queensland Animal Ethics Committee, under the guidelines of the National Health and Medical Research Council of Australia.

### EDiPS construct

The TH + GCH1 (EDiPS) construct contains the human tyrosine hydroxylase (huTH) and human GTP cyclohydrolase 1 (huGCH1) genes, each driven by a human synapsin-1 promoter (Fig. 1a). The control construct was the huGCH1 cassette alone, and driven by a chicken beta-actin (CBA) promoter. Trial experiments revealed an adeno-associated viral (AAV1) vector was optimal to deliver these constructs. The viral titre was 6.7 × 10^12^ gc/ml for the EDiPS and control viruses.

### Construct injections

The AAV-packaged construct was stereotactically delivered to the SNpc under isoflurane anaesthesia (4% during induction, maintained at 1.5–2%). Animals were injected bilaterally at (from bregma): A-P: −5.2 mm, M-L: +/−2.0 mm, D-V (from dura): −7.6 mm. One microlitre of the EDiPS virus, control virus or vehicle (saline) was delivered using a pulled glass capillary injector attached to a syringe. After 48 h of isolated recovery, animals were returned to pair-housing until behavioural testing approximately 6 weeks later. A proof-of-concept study was conducted with a unliateral injection to visualize the increase in TH production induced by the EDiPS construct (Fig. 2a).

### AMPH-induced locomotion

AMPH-induced locomotion was assessed in matt black 60 × 60 × 60 cm chambers. Baseline locomotion was recorded for 30 min, then animals were removed and injected with 0.6 mg/kg of dexamphetamine intraperitoneally (i.p.). Animals were then placed back into the chamber and recorded for a further 2 h. Distance travelled was calculated using EthoVision software (Noldus, Ver. 13.0).

### Pre-pulse inhibition

The suppression of the startle response was measured by placing rats into clear Plexiglass cylinders on a platform housed in sound attenuating chambers controlled using specialist software (SR-Lab, San Diego Instruments). Briefly, pre-pulses at three different intensities (74, 78, 86 dB) were played at a variety of intervals (8, 16, 32, 64, 128, 256 ms) prior to the startle pulse (120 dB) to assess PPI. The median of each trial type was used for analysis.

### Quinpirole-induced locomotion

Quinpirole-induced locomotion was assessed and analysed with the same protocol as for AMPH-induced locomotion. Animals were first tested with 0.05 mg/kg, and placed into the chamber immediately following the i.p. injection and recorded for 1 h. A week later, animals were tested with 0.5 mg/kg. For this test, animals were first habituated to the chamber for 30 min, then removed and the i.p. injection was performed. Animals were then placed back into the chamber, recorded for a further 90 min and locomotion was assessed.

### Microdialysis

Microdialysis was performed under isoflurane anaesthesia. The probe (CMA 12 Elite, Harvard Apparatus) positions were DS = AP: +0.6, LM: −2.6 DV: −5.0, NAc = AP: +1.6, LM: −0.9, DV: −7.2, PFC = AP: +3.2, LM: −0.6, DV: −5.0. Artificial CSF (aCSF) was perfused at a rate of 1ul/min. Samples were collected every 20 min into tubes containing 5 μl of 0.1 M perchloric solution. Following baseline acquisition, AMPH (0.6 mg/kg) was injected i.p. and six samples were collected over two hours. Finally, a 100 μm KCl solution was exchanged for the normal aCSF perfusate, and an additional 3 × 20 min samples were collected. Following microdialysis, brain sections were stained with cresyl violet to assess probe placements (Fig. S7).

### Monoamine assessment

Dialysate (15 μl) was injected into an Agilent 1200 Series HPLC system (Agilent Technologies, Inc., CA, USA). The mobile phase was 12% acetonitrile, 25 mM NaH_2_PO_4,_ 50 mM C_6_H_8_O_7_.H_2_O, 1.4 mM Octane and 1 mM EDTA, pH adjusted to 4.15 with NaOH, delivered isocratically at a flow of 0.6 ml/min to a SunFire C18 3.0 mm × 100 mm × 3.5 μm column (Waters). DA, 5-HT (5-hydroxytryptamine; serotonin) and their metabolites were detected using a Coulochem III electrochemical detector (ESA Laboratories, Inc., MA, USA). The analytic cell (Model 5014B) was set to +250 mV (ESA Laboratories, Inc., MA, USA). Data were quantified by calculating peak-height ratios for each analyte relative to the internal standard, deoxyepinephrine, and ratios compared with that from a standard curve.

### ^1^H-MRS

Proton magnetic resonance spectra (^1^H-MRS) were collected under isoflurane anesthesia using a Bruker BioSpec 9.4T MRI scanner. An 86 mm quadrature resonator was used for RF transmission, and a four-channel phased array rat head coil for signal detection. A point-resolved spectroscopy sequence (PRESS) was used to obtain water-suppressed metabolite spectra with the following parameters: TE = 9.9 ms; TR = 2500 ms; averages = 256. Spectra were acquired sequentially from three regions (DS, NAc and PFC), as shown in Fig. 5a. The voxel dimensions were; DS: 3 × 2 × 3 mm, NAc: 6 × 2 × 2 mm and PFC: 3 × 2 × 2 mm. Prior to AMPH administration, baseline scans of each region were acquired. Next, AMPH was delivered i.p. and 20 min spectra were acquired from the same regions in order. Spectra were analysed using Linear Combination of Model spectra software (LCModel, version 6.3) using a reference basis set with the same data acquisition parameters. Metabolites with a %SD > 20 were rejected from the analysis. The difference between pre- and post-AMPH values were calculated for each animal, and the mean delta compared between groups.

### Immunohistochemistry

Immunohistochemistry was used to confirm construct expression both at the injection site (SNpc) and the DS target. Immunohistochemistry was performed using primary antibodies raised against TH (MAB318, monoclonal mouse IgG, 1:1000, Millipore) and huGCH1 (HPA028612, polyclonal rabbit IgG, 1:250, Sigma), and secondaries Alexa Fluor 488 goat anti-rabbit, and Alexa Fluor 568 goat anti-mouse (Life Technologies, 1:500). The proof-of-concept unilaterally injected animals were sectioned at 40 μm, and a series encompassing the striatum and midbrain were stained for TH. The posterior brain portions of cohort 1 were sectioned at 40 μm, in a 1-in-6 series, and stained for TH and huGCH1.

### RNA extraction, cDNA synthesis, qPCR

Total RNA was extracted using an RNeasy Micro Kit (Qiagen) and cDNA synthesis was then performed using the SuperScript IV Reverse Transcriptase kit (Invitrogen). mRNA levels of genes of interest—see Table S5 for all primers—were measured using SensiFAST^TM^ SYBR® No-ROX. Gene expression was established relative to the housekeeping gene, glyceraldehyde 3-phosphate dehydrogenase (GAPDH). HuTH expression was also quantified. The proportion of huTH by region was calculated by establishing the relative level of mRNA corrected for the average weight of tissue of that region.

### Statistics

Results were analysed using SPSS software (ver. 24, SPSS Inc., Chicago, Illinois). Independent one-sided *t*-tests were used for simple comparisons between groups. When multiple *t*-tests were performed, a Bonferroni adjustment was performed to account for multiple comparisons. A MANOVA was used to compare between groups when multiple analytes were assessed from multiple regions. Otherwise ordinary or repeated measures (RM) ANOVA were used where appropriate. Significant effects were followed up with independent *t*-tests. The significance level was *p* < 0.05.

### Reporting Summary

Further information on experimental design is available in the Nature Research Reporting Summary linked to this article.

## Supplementary information

Reporting Summary

Supplementary Figures and Methods

## Data Availability

The datasets generating during this study are available from the corresponding author on reasonable request.

## Supplementary information

**Supplementary information** accompanies the paper on the *npj Schizophrenia* website (10.1038/s41537-019-0074-z).
