# Supplementary material for: Enhanced Dopamine in Prodromal Schizophrenia (EDiPS): a new animal model of relevance to schizophrenia
Source: NPJ Schizophr. 2019 Mar 29;5:6. doi: 10.1038/s41537-019-0074-z (PMC6441087; doi:10.1038/s41537-019-0074-z)
Supplement: Supplementary file 2 — Supplementary Figures and Methods [file 41537_2019_74_MOESM2_ESM.docx]

**Supplementary Figures**

Supplementary Figure 1:

Figure S1. Association between ratTH and huTH in EDiPS animals. There was no significant association between the amount of huTH and the amount of ratTH in either the dorsal medial striatum (DMS; p<0.09) or the dorsal lateral striatum (DLS; p<0.5). This indicates that the increase in huTH in EDiPS animals has not resulted in a compensatory change in the amount of endogenous ratTH.

Supplementary Figure 2:

a)

b)

c)

d)

e)

f)

g)

h)

i)

Figure S2. Microdialysis results for dopamine metabolites in the three regions. BL indicates the average of three baseline collection points. AMPH is delivered at the start of the first AMPH timepoint (A1), and 6 AMPH timepoints are collected (A1-6). Each timepoint represents a 20-minute bin. When DOPAC (a) is assessed with a repeat measures ANOVA, there is a time*EDiPS interaction in the DS (*F*_(6,66)_=2.32, *p*=0.04), with EDiPS animals showing a significant decrease in DOPAC at the baseline timepoint (*p*=0.047). There were no significant differences for DOPAC in the NAc (c) or PFC (e), or for the other metabolites HVA (b,d,f) or 3-MT (g,h,i). *= p<0.05. **DOPAC:** 3,4-dihydroxyphenylacetic acid. **HVA:** homovanillic acid. **3-MT:** 3-methoxytyramine. DS n=6 (control), n=7 (EDiPS); NAc n=7 (control), n=6 (EDiPS); PFC n=7 (control), n=8 (EDiPS). ±SEM

Supplementary Figure 3:

a)

b)

c)

d)

e)

f)

Figure S3. Microdialysis results for 5-HT (serotonin) and its metabolite 5-HIAA. BL indicates the average of three baseline collection points. Amphetamine (AMPH) is delivered at the start of the first AMPH timepoint (A1), and 6 AMPH timepoints are collected (A1-6). Each timepoint represents a 20-minute bin. There was an effect of EDiPS on 5-HT (a) in the DS (*F*_(6,66)_=4.85. *p*=0.049) which was significantly increased at baseline (*p*=0.042), as well as at A2 (*p*=0.044), compared to controls. There were no significant differences for 5-HT in the NAc (c) or PFC (e), or for 5-HIAA in any region (b,d,f). *= p<0.05. **5-HT:** 5-hydroxytryptamine (serotonin), **5-HIAA:** 5-hydroxyindoleacetic acid. DS n=6 (control), n=7 (EDiPS); NAc n=7 (control), n=6 (EDiPS); PFC n=7 (control), n=8 (EDiPS). ±SEM.

Supplementary Figure 4:

Ai)

Aii)

Bii)

Bi)

Ci)

Cii)

Di)

Dii)

*R^2^*=0.05

*p*=0.62

*R^2^*=0.04

*p*=0.63

*R^2^*=0.51

*p*=0.11

*R^2^*=0.57

*p*=0.08

*R^2^*=0.005

*p*=0.87

*R^2^*=0.22

*p*=0.27

*R^2^*=0.03

*p*=0.73

*R^2^*=0.02

*p*=0.74

a)

b)

c)

d)

e)

f)

g)

h)

Figure S4. Correlations between midbrain construct expression and DA release. Correlations between AMPH- (a) and KCl (b)-induced DS DA release and the number of HuGCH1+ cells in the substantia nigra (SN). Correlations between AMPH- (c) and KCl (d)-induced NAc DA release and the number of HuGCH1+ cells in the ventral tegmental area (VTA). Correlations between AMPH- (e) and KCl (f)-induced DS DA release and the intensity of TH fluorescence in the SN. Correlations between AMPH- (g) and KCl (h)-induced NAc DA release and the intensity of TH fluorescence in the VTA. No statistically significant correlations were evident for any association.

Supplementary Figure 5:

a)

b)

Figure S5. Correlations between AMPH- and KCl-induced DA release, in the dorsal striatum (a) and nucleus accumbens (b). Pearson coefficients were calculated, and indicate that in the DS, there was no correlation between AMPH- and KCl-induced DA release for either control animals (*R^2^*=0.18, *p*=0.40) or EDiPS animals (*R^2^*=0.22, *p*=0.28). Similarly, in the NAc there was no correlation for either control animals (*R^2^*=0.25, *p*=0.26) or EDiPS animals (*R^2^*=0.57, *p*=0.08).

Supplementary figure 6:

A)

a)

b)

Figure S6. Behavioural data with the vehicle (PBS) and control (AAV-GCH1) injected groups separated. Data is displayed for AMPH-induced hyperlocomotion (a) and pre-pulse inhibition (b). There are no significant differences between the control groups, and therefore these groups were pooled for all analyses. **p*<0.05. ±SEM

Supplementary Figure 7:

Figure S7. Microdialysis probe positions included for analysis for the dorsal striatum, nucleus accumbens and prefrontal cortex.

**Supplementary tables**

Supplementary table 1. HuGCH1 staining in regions of the midbrain for cohort 1.

| **Animal** | Left hemisphere | | | Right hemisphere | | |
| --- | --- | --- | --- | --- | --- | --- |
|  | **SNpc** | **SNr** | **VTA** | **SNpc** | **SNr** | **VTA** |
| 1 | - | - | - | + | + | + |
| 2 | + | + | + | + | + | + |
| 3 | + | + | - | + | + | - |
| 4 | + | - | + | + | + | + |
| 5 | + | + | + | + | + | + |
| 6 | - | - | - | + | - | - |
| 7 | + | + | - | - | - | - |
| 8 | + | - | - | + | + | - |
| 9 | + | - | + | + | + | + |
| 10 | + | - | - | - | - | - |
| 11 | + | + | - | + | + | - |
| 12 | + | + | + | + | - | - |

HuGCH1 staining in the midbrain, for each of the 12 cohort 1 EDiPS animals, for each hemisphere. + : presence of huGCH1 staining, - : absence of huGCH1 staining; **SNpc:** substantia nigra pars compacta; **SNr:** substantia nigra pars reticulata; **VTA:** ventral tegmental area.

Supplementary table 2. mRNA levels for genes analysed. Mean (SEM).

| **Gene** | **Region** | **Control** | **EDiPS** |
| --- | --- | --- | --- |
| **huTH** | Lateral DS | n.d. | 0.00239 (0.0006) |
|  | Medial DS | n.d. | 0.00195 (0.0008) |
|  | NAc | n.d. | 0.000818 (0.0002) |
|  | PFC | n.d. | 0.000259 (0.00006) |
| **ratTH** | Lateral DS | 0.0195 (0.0.002) | 0.0219 (0.003) |
|  | Medial DS | 0.135 (0.009) | 0.142 (0.02) |
|  | NAc | 1.02 (0.08) | 0.868 (0.2) |
|  | PFC | 0.0210 (0.001) | 0.0238 (0.002) |
| **D1R** | Lateral DS | 1.46 (0.09) | 1.47 (0.2) |
|  | Medial DS | 1.34 (0.09) | 1.25 (0.06) |
|  | NAc | 1.93 (0.1) | 1.80 (0.2) |
|  | PFC | 0.0963 (0.007) | 0.0837 (0.007) |
| **D2S** | Lateral DS | 0.874 (0.06) | 1.07 (0.2) |
|  | Medial DS | 0.683 (0.04) | 0.616 (0.05) |
|  | NAc | 0.577 (0.04) | 0.504 (0.05) |
|  | PFC | 0.0179 (0.001) | 0.0155 (0.001) |
| **D2L** | Lateral DS | 1.79 (0.1) | 2.09 (0.3) |
|  | Medial DS | 1.45 (0.09) | 1.30 (0.09) |
|  | NAc | 1.20 (0.09) | 1.06 (0.1) |
|  | PFC | 0.0387 (0.003) | 0.0338 (0.003) |
| **D2T** | Lateral DS | 1.14 (0.06) | 1.37 (0.2) |
|  | Medial DS | 0.930 (0.06) | 0.819 (0.06) |
|  | NAc | 1.01 (0.09) | 0.922 (0.08) |
|  | PFC | 0.0183 (0.001) | 0.0164 (0.002) |
| **COMT** | Lateral DS | 0.182 (0.02) | 0.205 (0.03) |
|  | Medial DS | 0.194 (0.02) | 0.176 (0.01) |
|  | NAc | 0.289 (0.03) | 0.280 (0.02) |
|  | PFC | 0.156 (0.02) | 0.126 (0.02) |
| **MAOA** | Lateral DS | 0.549 (0.02) | 0.594 (0.06) |
|  | Medial DS | 0.518 (0.03) | 0.488 (0.03) |
|  | NAc | 0.771 (0.04) | 0.766 (0.04) |
|  | PFC | 0.623 (0.04) | 0.644 (0.07) |
| **VMAT** | Lateral DS | 0.000872 (0.00006) | 0.00109 (0.0002) |
|  | Medial DS | 0.00301 (0.0009) | 0.00456 (0.002) |
|  | NAc | 0.00384 (0.001) | 0.00772 (0.003) |
|  | PFC | 0.000325 (0.00006) | 0.000307 (0.00007) |
| **DAT** | Lateral DS | 0.00327 (0.0002) | 0.00334 (0.0005) |
|  | Medial DS | 0.000934 (0.00008) | 0.000907 (0.00009) |
|  | NAc | 0.000836 (0.0001) | 0.000908 (0.00009) |
|  | PFC | 0.00146 (0.0001) | 0.00150 (00.0001) |
| **AADC** | Lateral DS | 0.0305 (0.003) | 0.0317 (0.005) |
|  | Medial DS | 0.0306 (0.002) | 0.0299 (0.002) |
|  | NAc | 0.0707 (0.004) | 0.0660 (0.005) |
|  | PFC | 0.0167 (0.002) | 0.0146 (0.002) |

n.d. = not detectable. **huTH**: human tyrosine hydroxylase, **ratTH**: rat tyrosine hydroxylase, **D1R**: dopamine 1 receptor, **D2S**: dopamine 2 receptor short, **D2L**: dopamine 2 receptor long, **D2RT**: dopamine 2 receptor total, **COMT**: catechol-O-methytransferase, **MAOA**: monoamine oxidase A, **VMAT**: vesicular monoamine transporter, **DAT**: dopamine transporter, **AADC**: amino acid decarboxylase,

Supplementary table 3. Baseline ^1^H-MRS analytes. Data provided as ratio to Cr+PCr. Mean (SEM).

| Analyte | **DS** | | **NAc** | | **PFC** | |
| --- | --- | --- | --- | --- | --- | --- |
|  | **Control** | **EDiPS** | **Control** | **EDiPS** | **Control** | **EDiPS** |
| Glu | 1.346 (0.05) | 1.283 (0.03) | 1.158 (0.02) | 1.237 (0.03) | 1.405 (0.09) | 1.43 (0.07) |
| Gln | 0.647 (0.03) | 0.580 (0.02) | 0.579 (0.02) | 0.623 (0.03) | 0.694 (0.05) | 0.703 (0.04) |
| Glx | 1.994 (0.06) | 1.863 (0.04) | 1.737 (0.03) | 1.86 (0.05) | 1.98 (0.1) | 2.02 (0.1) |
| GSH | 0.259 (0.01) | 0.282 (0.01) | 0.235 (0.008) | 0.256 (0.008) | n.d. | n.d. |
| Ins | 0.379 (0.03) | 0.413 (0.02) | 0.673 (0.02) | 0.698 (0.02) | 0.5771 (0.02) | 0.599 (0.03) |
| GABA | 0.338 (0.02) | 0.345 (0.01) | 0.429 (0.02) | 0.434 (0.02) | n.d | n.d |
| NAA | 0.938 (0.05) | 0.965 (0.05) | 0.931 (0.01) | 0.942 (0.03) | 0.970 (0.04) | 1.008 (0.03) |
| Tau | 0.837 (0.06) | 0.822 (0.05) | 0.815 (0.03) | 0.863 (0.02) | 0.778 (0.04) | 0.791 (0.01) |
| GPC+PCh | 0.238 (0.01) | 0.245 (0.006) | 0.285 (0.008) | 0.280 (0.008) | 0.200 (0.006) | 0.216 (0.007) |
| NAA+NAAG | 1.141 (0.05) | 1.165 (0.05) | 1.039 (0.01) | 1.089 (0.02) | 1.113 (0.06) | 1.145 (0.05) |

**Glu**: glutamate, **Gln**: glutamine, **Glx:** glutamate+glutamine**, GSH**: glutathione, **Ins**: myo-inositol, **GABA**: gamma-aminobutyric acid**, NAA**: N-acetylaspartate, **Tau**: taurine, **GPC+PCh:** glycerophospocholine + phosphocholine, **NAA+NAAG:** N-acetylaspartate+N-acetylaspartylglutamate. Underlined = region effect.

Supplementary table 4. Delta values for ^1^H-MRS analytes. Delta calculated as post-AMPH Cr+PCr value minus pre-AMPH value. Mean (SEM).

| Analyte | **DS** | | **NAc** | | **PFC** | |  |
| --- | --- | --- | --- | --- | --- | --- | --- |
|  | **Control** | **EDiPS** | **Control** | **EDiPS** | **Control** | **EDiPS** | |
| Glu | -0.12 (0.05) | -0.055 (0.05) | -0.022 (0.03) | -0.086 (0.02) | -0.038 (0.1) | 0.038 (0.07) | |
| Gln | -0.047 (0.03) | 0.10 (0.04)* | 0.057 (0.03) | 0.0095 (0.02) | 0.011 (0.03) | -0.10 (0.05) | |
| Glx | -0.17 (0.06) | 0.047 (0.07)* | 0.035 (0.05) | -0.076 (0.04) | 0.071 (0.1) | 0.039 (0.07) | |
| GSH | 0.031 (0.02) | 0.012 (0.02) | -0.009 (0.04) | -0.0037 (0.009) | n.d | n.d. | |
| Ins | 0.007 (0.05) | 0.059 (0.02) | 0.025 (0.03) | 0.008 (0.02) | 0.026 (0.05) | 0.020 (0.03) | |
| GABA | n.d. | -0.027 (0.03) | 0.054 (0.02) | -0.002 (0.03) | n.d. | n.d. | |
| NAA | 0.12 (0.08) | -0.048 (0.06) | 0.028 (0.01) | -0.0004 (0.02) | 0.0087 (0.04) | 0.034 (0.03) | |
| Tau | 0.052 (0.04) | 0.091 (0.03) | -0.039 (0.03) | -0.023 (0.02) | -0.010 (0.05) | -0.031 (0.04) | |
| GPC+PCh | -0.024 (0.006) | -0.011 (0.008) | 0.001 (0.005) | -0.0036 (0.005) | -0.016 (0.006) | -0.029 (0.01) | |
| NAA+NAAG | 0.006 (0.07) | -0.013 (0.05) | 0.025 (0.02) | -0.032 (0.02) | 0.023 (0.05) | 0.029 (0.02) | |

**Glu**: glutamate, **Gln**: glutamine, **Glx:** glutamate+glutamine**, GSH**: glutathione, **Ins**: myo-inositol, **GABA**: gamma-aminobutyric acid**, NAA**: N-acetylaspartate, **Tau**: taurine, **GPC+PCh:** glycerophospocholine + phosphocholine, **NAA+NAAG:** N-acetylaspartate+N-acetylaspartylglutamate. *= sig. different to controls.

Supplementary Table 5. Primers used for qPCR

| **Target gene** | **Direction** | **Sequence 5’–3’** |
| --- | --- | --- |
| Rat GAPDH | Forward | ATCCTGCACCACCAACTGCT |
|  | Reverse | GGGCCATCCACAGTCTTCTG |
| Human TH | Forward | CTGTGAAGGTGTTTGAGACGT |
|  | Reverse | CTTGTCCAGCTCTGACACTTT |
| Rat TH | Forward | AGTACAAGCACGGTGAACCA |
|  | Reverse | GATGCTGTCCTCTCGGTAGC |
| Rat D1R | Forward | GGACACCGAGGATGACAACT |
|  | Reverse | TGGCTACGGGGATGTAAAAG |
| Rat D2R short | Forward | TGCTGGTCTATATCAAAATCTACATCG |
|  | Reverse | GGCAGCATCCTTGAGTGGTGT |
| Rat D2R long | Forward | AGCAGTCGAGCTTTCAGAGCC |
|  | Reverse | ATTCTCCGCCTGTTCACTGG |
| Rat D2R total | Forward | CCCAGAGAGGACCCGGTATAG |
|  | Reverse | CTGGTTTGGCAGGACTGTCA |
| Rat DAT | Forward | GGGTTTGGAGTGCTGATTGC |
|  | Reverse | GACGACGAAGCCAGAGGAGA |
| Rat VMAT2 | Forward | CGCAAACTG ATCCTGTTCAT |
|  | Reverse | AGAAGATGCTTTCGGAGGTG |
| Rat AADC | Forward | TGGTTGTCACCCTAGGAACC |
|  | Reverse | GCATCAATGTGCAGCCATAC |
| Rat MAOA | Forward | GCCAGGAACGGAAATTTGTA |
|  | Reverse | TCTCAGGTGGAAGCTCTGGT |
| Rat COMT | Forward | ATCTTCACGGGGTTTCAGTG |
|  | Reverse | GAGCTGCTGGGGACAGTAAG |
| Human GCH1 | Forward | CGCCTACTCGTCCATCCATCCTGA |
|  | Reverse | CCTTCACAATCACCATCTCA |

**GAPDH:** glyceraldehyde 3-phosphate dehydrogenase. **TH:** tyrosine hydroxylase. **D1R:** dopamine receptor 1. **D2R:** dopamine receptor 2. **DAT:** dopamine transporter. **VMAT2:** vesicular monoamine transporter 2. **AADC:** amino acid decarboxylase. **MAOA:** monoamine oxidase A. **COMT:** catechol-O-methyltransferase. **GCH1:** GTP cyclohydrolase 1.

Supplementary methods:

*Pre-pulse inhibition (PPI):* The suppression of the startle response was measured by placing rats into a clear Plexiglass cylinder on a platform housed in sound attenuating chambers controlled using specialist software (SR-Lab, San Diego Instruments). The test session consisted of the pseudo-randomized presentation of the different trial types. The acoustic startle response (ASR) was measured using a single 40ms pulse at various intensities (70, 80, 90, 100, 110, 120dB) and habituation was measured as the change in startle response to a 110dB pulse presented at the start, middle and end of the session. Pre-pulses at three different intensities (74, 78, 86Hz) were played at a variety of intervals (8, 16, 32, 64, 128, 256ms) prior to the startle pulse (120dB) to assess pre-pulse inhibition. Each trial type was presented five times, and the median was used for analysis. Percentage PPI was calculated as: [(startle amplitude of ASR trial – startle amplitude on pre-pulse trial)/startle amplitude of ASR trial] x100.

*Tissue collection:*  The proof-of-concept unilaterally injected cohort were transcardially perfused with 4% PFA 6 weeks following the viral injection. Cohort 1 was perfused one week following the final behavioural test. The extracted brains were divided into the forebrain and midbrain coronally. The right forebrain was divided into medial and lateral DS portions, NAc and PFC, and all regions were used for the expression of DA-related genes. The posterior half of the brain was post-fixed in 4% PFA overnight. Cohort 2 was immediately perfused with 4% PFA following microdialysis. Cohort 3 was perfused with 4% PFA one week following ^1^H-MRS.

*RNA extraction, cDNA synthesis, qPCR*: Following tissue homogenization in Qiazol, total RNA was extracted using an RNeasy Micro Kit (Qiagen). cDNA synthesis was then performed using the SuperScript IV Reverse Transcriptase kit (Invitrogen). The cDNA was diluted 1:10 for use in the qPCR reactions for huTH, VMAT2 and DAT, and diluted 1:30 for ratTH, D1R, D2RS, D2RL, D2RT, AADC, COMT, and MAOA. mRNA levels of genes of interest were measured using SensiFAST^TM^ SYBR® No-ROX in a 384-well format. Gene expression was established relative to the housekeeping gene, glyceraldehyde 3-phosphate dehydrogenase (GAPDH). HuTH was only detectable in EDiPS animals. Proportion of huTH by region was calculated by mRNA in proportion to the average weight of tissue of that region. All relevant primer sequences are in supplementary Table 1. Non-template-controls were included on each PCR plate. The reaction conditions were as follows: 95°C for 5 minutes, then 40 cycles of 95°C for 10 seconds, 60°C for 10 seconds and 72°C for 20 seconds.

*Microdialysis surgery:* Anaesthetized microdialysis surgery was performed. Briefly, the 3mm DS probe (CMA 12 Elite, Harvard Apparatus) was implanted at AP: +0.6, LM: -2.6 DV: -5.0 and cemented in place. The PFC probe (4mm length) was implanted at AP: +3.2, LM: -0.6, DV: -5.0, and cemented into place. The NAc probe (2mm length) was positioned at AP: +1.6, LM: -0.9, DV: -7.2, and held in place using the probe holder for the duration of the experiment. Artificial CSF (aCSF) was perfused over the membrane at a rate of 1ul/min. Samples were collected every 20 mins into pre-weighed tubes containing 5ul of 0.1M perchloric solution. Following 100 minutes of baseline acquisition, AMPH (0.6mg/kg) was injected i.p. and 6 samples were collected over two hours. Finally, a 100um KCl solution was exchanged for the normal aCSF perfusate, and an additional 3 samples were collected. Animals were perfused with 4% PFA following the surgery, and brains sectioned to assess probe placement.

*Semi-quantitative immunohistochemical analysis:*

In order to correlate midbrain construct expression with DA release in the forebrain regions, staining for TH and huGCH1 was performed on midbrain sections from the microdialysis cohort. A 1-in-5 series of sections through the midbrain was stained using primary antibodies raised against TH (MAB318, monoclonal mouse IgG, 1:1000, Millipore) and huGCH1 (HPA028612, polyclonal rabbit IgG, 1:250, Sigma), and secondaries Alexa Fluor 488 goat anti-rabbit, and Alexa Fluor 568 goat anti-mouse (Life Technologies, 1:500). The sections were then assessed by eye, and the section with the greatest amount of huGCH1 staining in the midbrain region was taken for further analysis. Images of these sections were taken (using the same exposure times for all sections) using an Axio Imager standard microscope. Two different analyses were then performed. First, TH immunofluorescence was assessed using Fiji software, to provide a pixel density of mean fluorescence. This was performed for the two regions of interest in the midbrain; the substantia nigra (SN; including the pars compacta and pars reticulata) and the ventral tegmental area (VTA). Secondly, Imaris software was used to count the number of huGCH1 “spots” in these two regions, to quantify the number of GCH1+ cells. These analyses were performed for each hemisphere, and the average values used for each animal. These values were compared to pooled DA release values for either AMPH- or KCl-induced DA release. Construct expression in the SN was compared with DA release in the DS, and expression in the VTA was compared with DA release in the NAc. Linear regression analyses were performed to determine if any significant correlations were present.

*^1^H-MRS:*

The ^1^H-MRS scanning was performed under isoflurane anaesthesia. Data acquisition was performed on a Bruker BioSpec 9.4T MRI scanner with Paravision 5.1/6.0 (Bruker, Germany). A baseline scan was obtained for each region in sequence (DS, then NAc, then PFC) and then AMPH was delivered i.p. and after 20 mins the same regions were scanned again in the same order (Fig. 5A). Three voxels were used; DS (unilateral): 3x2x3mm, NAc (bilateral): 6x2x2mm and PFC (bilateral): 3x2x2mm (Fig. 5A). A non-suppressed reference water signal from each voxel was obtained for assessment of linewidths and as a metabolite concentration reference. A point-resolved spectroscopy sequence (PRESS) was used to obtain water-suppressed metabolite spectra with the following parameters: TE – 9.9 ms; TR – 2500 ms; averages – 256. ^1^H MRS data were processed on TOPSPIN and analysed in Linear Combination of Model spectra (LCModel version 6.3-1J) software, using a reference basis set with the same data acquisition parameters. Metabolites with a %SD >20 were rejected from the analysis. The concentration of individual metabolites was expressed as a ratio to total creatine (Cr+PCr). To compare the effect of AMPH, the difference between pre- and post-AMPH values were calculated for each animal, and the mean delta compared between groups. Significant differences between mean delta values were further examined with paired *t*-tests.
